# Optimization of electrocoagulation process to treat grey wastewater in batch mode using response surface methodology

**DOI:** 10.1186/2052-336X-12-29

**Published:** 2014-01-10

**Authors:** Thirugnanasambandham Karichappan, Sivakumar Venkatachalam, Prakash Maran Jeganathan

**Affiliations:** 1 Department of Food Technology, Kongu Engineering College, Perundurai Erode-638052, TN India; 2 Department of Food Technology, Kongu Engineering College, Perundurai Erode-638052, TN India; 3 Department of Food Technology, Kongu Engineering College, Perundurai Erode-638052, TN, India

**Keywords:** Electrocoagulation, Grey wastewater, Stainless steel electrodes, Box-Behnken design, Mathematical model

## Abstract

**Background:**

Discharge of grey wastewater into the ecological system causes the negative impact effect on receiving water bodies.

**Methods:**

In this present study, electrocoagulation process (EC) was investigated to treat grey wastewater under different operating conditions such as initial pH (4–8), current density (10–30 mA/cm^2^), electrode distance (4–6 cm) and electrolysis time (5–25 min) by using stainless steel (SS) anode in batch mode. Four factors with five levels Box-Behnken response surface design (BBD) was employed to optimize and investigate the effect of process variables on the responses such as total solids (TS), chemical oxygen demand (COD) and fecal coliform (FC) removal.

**Results:**

The process variables showed significant effect on the electrocoagulation treatment process. The results were analyzed by Pareto analysis of variance (ANOVA) and second order polynomial models were developed in order to study the electrocoagulation process statistically. The optimal operating conditions were found to be: initial pH of 7, current density of 20 mA/cm^2^, electrode distance of 5 cm and electrolysis time of 20 min.

**Conclusion:**

These results indicated that EC process can be scale up in large scale level to treat grey wastewater with high removal efficiency of TS, COD and FC.

## Background

With growing urbanization and rapid industrialization, the problem of the release of untreated wastewater into the ecosystem has been of increasing concern in many parts of the world. Since wastewaters can significantly contaminate the receiving water bodies, which cannot be ignored any longer. Therefore, the removal of toxic pollutants from wastewaters has recently become the subject of considerable interest due to more strict legislations introduced in many countries to control water pollution [1]. Grey wastewater has been recognized, one of the wastewater which includes water from baths, showers, hand basins, washing machines, dishwashers, kitchen sinks and constitutes 50–80% of the total household wastewater, but excludes streams from toilets. The discharges of untreated grey wastewater in the ecosystem have substantial impacts on the environment and human health [2]. Numerous studies have been conducted on the treatment of grey wastewater with different technologies which include UASB reactor [3], sequence batch reactor [4], membrane bioreactor [5] and vertical flow constructed wetlands [6]. These methods have been practiced as the prime method to treat grey wastewater for many years. However, these methods leads to a special problem of sludge handling, removal efficiency and their high costs limit their use in practice. So there is a critical need to develop more efficient and inexpensive method to treat grey wastewater.

In recent years, investigations have been focused on the treatment of grey wastewater using electrocoagulation (EC) process which offers several advantages including ease of operation, robustness to varying reaction conditions and effluent types, less retention time, rapid sedimentation of the electrogenerated flocculants, less sludge production, and smaller space requirements and capital costs [7]. In addition, EC process has been applied to treat various wastewaters such as electroplating wastewaters [8], paper mill bleaching wastewater [9], chemical mechanical polishing wastewater [10], textile wastewater [11] and olive oil wastewater [12]. Extensive literature survey shows that none of researchers studied the optimization of the EC process using a stainless steel electrode to treat grey wastewater. To date, most studies on the optimization of wastewater treatment process have focused on the traditional one-factor-at-a-time approach. However, this approach, which does not take into account cross effects from the factors considered, is time consuming and results in poor optimization results [13]. Response surface methodology (RSM) is a powerful statistical-based technique for modeling complex systems, evaluating the simultaneous effects of several factors, and thus searching optimum conditions for desirable responses [14]. RSM also generates a mathematical model that can be used to predict the response of a system to any new condition. However, still now, RSM has not been used as a modeling and optimization tool for EC process to treat grey wastewater in batch mode. Hence, in this study, Box-Behnken response surface design (BBD) coupled with Derringer’s desired function methodology was used to optimize and investigate the influence of the key process variables of EC such as initial pH, current density, electrode distance and electrolysis time (independent variables) on total solids (TS) removal, chemical oxygen demand (COD) removal and fecal coliforms (FC) removal (dependent variables).

## Materials and methods

### Wastewater sample and chemicals

The wastewater used in this study was collected from a tank containing a mixture of domestic wastewater in Erode, TamilNadu, southern India and were stored at 4°C prior to the experiments. The composition of the wastewater is shown in Table 1. All reagents were of analytical grade and were used without further purification. Solutions were prepared at room temperature with deionised water.

**Table 1:** Composition of grey wastewater

**Composition of grey wastewater**	
pH	5.78
TS (mg/L)	258
COD (mg/L)	646
Fecal coliform (CFU × 10^5^/mL)	2.2

### Experimental setup and procedure

**Figure 1:**
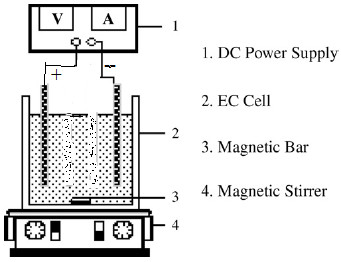
Schematic diagram of electrocoagulation (EC) process unit.

Acrylic made tank having working volume of about 3 L was used to conduct the EC experiments. Stainless steel sheets of 33 cm × 6 cm were used as electrodes for EC process. The gap between the anode and cathode was varied from 4 to 6 cm. The entire electrode assembly was fitted on non-conducting wedges and hanged from the top of the electrocoagulation tank. The effective surface area of each electrode was 108 cm^2^. The assembly was connected to DC power source (Dolphin: 0–6 A and 0–30 V) to fix the desired current density. Schematic diagram of electrocoagulation reactor is shown in Figure 1. In each run, 1.6 L wastewater was placed into the reactor and all the runs were performed at constant stirring speed of 250 rpm and l g/L of NaCl as a supporting electrolyte. After the EC process, the power was switched off and the electrodes were dismantled. Before each run, impurities on the SS electrode surfaces were removed by dipping for 5 min in acetone solution. The treated wastewater collected at different time interval was filtered and used for analysis. Before analysis, treated wastewater was centrifuged at 6500 rpm for 15 min (Remi R-24 Centrifuge, India) and supernatant liquid were collected for the determination of TS, COD and FC.

### Analytical methods

Measurements of initial pH, total solids (TS), chemical oxygen demand (COD) and fecal coliform (FC) were done in accordance with the standard methods reported in elsewhere [15]. The removal efficiency (R) was calculated using the following equation [16]

(1) $$R\;\left(\%\right)=\frac{Y_{0}-Y}{Y}\times 100$$

where Y_0_ and Y represent are the initial and final value of TS, COD and FC.

### Experimental design

In this study, Box-Behnken response surface experimental design (BBD) with four factors at five levels was used to optimize and investigate the influence of process variables such as initial pH (4–8), current density (10–30 mA/cm^2^), electrode distance (4–6 cm) and electrolysis time (5–25 min) on the TS, COD and FC removal. Process variables and their ranges were determined based on the single factor experimental analysis. After selection of process (independent) variables and their ranges, experiments were established based on a BBD and the complete design consists of 29 experiments with five centre points.

A second-order polynomial equation was fitted to correlate the relationship between independent variables and responses, which accounts for variations caused by linear, quadratic and interactive effect of the process variables [17]. All the statistical analyses were done with the help of Stat ease Design Expert 8.0.7.1 statistical software package (Stat-Ease Inc., Minneapolis, USA). The details of methodology used for analyzing the experimental data and model are given in elsewhere [18].

## Results and discussions

Electrocoagulation process using stainless steel electrodes to treat gery wastewater was carried out at different initial pH, current density, electrode distance and electrolysis time to investigate the removal efficiency of TS, COD and FC. In this study, four factors with five levels BBD was used to evaluate the effect and optimize the process variables on the responses. A total number of 29 batch experiments including five centre points were carried out in triplicates using statistically deigned experiments and the results are shown in Table 2.

**Table 2:** Box-Behnken experimental design matrix with experimental and predicted response values

**Initial pH (X**_**1**_**)**	**Current density (X**_**2**_**)**	**Electrode distance (X**_**3**_**)**	**Electrolysis time (X**_**4**_**)**	**TS removal (Y**_**1**_**)**	**COD removal (Y**_**2**_**)**	**FC removal (Y**_**3**_**)**
8	30	5	15	74.69	69.54	77.58
4	30	5	15	55.78	48.58	54.36
6	30	5	5	48.68	41.58	45.68
4	20	6	15	30.75	29.58	32.58
6	20	5	15	92.56	88.65	90.56
6	30	5	25	95.65	90.54	91.05
6	20	5	15	92.56	88.65	90.56
6	20	4	25	95.56	92.56	93.56
6	10	6	15	40.78	36.98	43.58
4	10	5	15	21.56	18.65	26.54
6	30	4	15	97.56	93.68	95.96
6	10	5	25	67.94	64.28	62.58
6	30	6	15	63.04	57.68	65.58
6	20	5	15	92.56	88.65	90.56
8	10	5	15	45.36	39.64	41.58
6	20	6	25	70.14	66.76	72.65
8	20	5	25	78.95	71.08	72.65
8	20	4	15	82.36	78.91	81.64
8	20	6	15	37.15	36.54	40.56
6	10	4	15	49.56	40.98	45.65
6	20	4	5	35.24	29.38	37.28
6	20	5	15	92.56	88.65	90.56
4	20	4	15	36.28	31.58	34.58
6	10	5	5	18.68	16.89	22.56
8	20	5	5	21.56	18.64	24.65
6	20	6	5	12.56	10.58	15.65
4	20	5	5	12.63	11.58	17.58
4	20	5	25	52.65	48.65	55.47
6	20	5	15	92.56	88.65	90.56

### Selection of suitable mathematical model

The experimental data was analyzed by model summary statistics in order to obtain regression models and decide about the adequacy of various models (linear, interactive, quadratic and cubic) to represent the EC process significantly. The results are listed in Table 3. From the Table 3, it was found that, linear and interactive (2FI) models shows lower coefficient of determination (R^2^), adjusted -R^2^, predicted -R^2^ and also having high *p*-values, when compared with quadratic model. Cubic model was found to be aliased. Therefore the quadratic model is chosen to describe the effects of operating variables on the EC process to treat grey wastewater [19]. Further more, analysis of variance (ANOVA) is also used to check the adequacy of quadratic model.

**Table 3:** Model summary statistics tested for the responses

**Model summary statistics**					
**Std. Dev.**	**R**^**2**^	**Adjusted R**^**2**^	**Predicted R**^**2**^	**PRESS**	**Remarks**
Total solids removal					
19.1689	0.6177	0.5539	0.5223	11017.7	
21.3110	0.6456	0.4487	0.3755	14403.9	
3.1819	0.9939	0.9877	0.9646	816.4340	Suggested
0.7371	0.9999	0.9993	0.9796	469.4592	Aliased
Chemical oxygen demand removal					
19.3375	0.5970	0.5298	0.4959	11224.9	
21.3946	0.6300	0.4244	0.3511	14449.5	
4.4728	0.9874	0.9748	0.9276	1613.2875	Suggested
1.5086	0.9994	0.9971	0.9117	1966.4244	Aliased
Fecal coliform removal					
18.3127	0.5985	0.5316	0.4954	10114.4	
20.2988	0.6300	0.4245	0.3381	13267.6	
4.8198	0.9838	0.9676	0.9065	1873.3270	Suggested
1.0705	0.9997	0.9984	0.9506	990.1164	Aliased

### Mathematical model fitting

The results obtained from BBD experiments were evaluated by multiple regression analysis method for EC process. An empirical relationship between the response and independent variables has been expressed by a second-order polynomial equation with interaction terms was fitted between the experimental results obtained on the basis of BBD, which will help to predict the efficiency of EC process in different sets of combinations. Three empirical models were developed to understand the interactive correlation between the responses and process variables. The final model obtained in terms of coded factors is given below

(2) $$\begin{array}{l} Y_{1}=92.56+10.87X_{1}+15.96X_{2}-1.1.85X_{3}\\ \;+25.96X_{4}-1.22X_{1}X_{2}-9.92X_{1}X_{3}+4.34X_{1}X_{4}\\ \;-6.44X_{2}X_{3}-0.57X_{2}X_{4}-0.69X_{3}X_{4}-29.44X_{1}^{2}\\ \;-13.25X_{2}^{2}-16.79X_{3}^{2}-21.88X_{4}^{2} \end{array}$$

(3) $$\begin{array}{l} Y_{2}=88.65+10.48X_{1}+15.35X_{2}-10.75X_{3}\\ \;+25.44X_{4}-\left(7.500E-0.003\right)X_{1}X_{2}-10.09X_{1}X_{3}\\ \;+3.84X_{1}X_{4}-8.00X_{2}X_{3}+0.39X_{2}X_{4}-1.75X_{3}X_{4}\\ \;-29.16X_{1}^{2}-14.65X_{2}^{2}-16.38X_{3}^{2}-21.71X_{4}^{2} \end{array}$$

(4) $$\begin{array}{l} Y_{3}=90.56+9.80X_{1}+15.64X_{2}-9.84X_{3}+23.71X_{4}\\ \;+2.04X_{1}X_{2}-9.77X_{1}X_{3}+2.53X_{1}X_{4}-7.08X_{2}X_{3}\\ \;+1.34X_{2}X_{4}+0.18X_{3}X_{4}-27.46X_{1}^{2}-13.34X_{2}^{2}\\ \;-1.5.02X_{3}^{2}-21.01X_{4}^{2} \end{array}$$

where, Y_1_, Y_2_ and Y_3_ are percentage of TS, COD, FC removal ; X_1_, X_2_, X_3_ and X_4_ are initial pH, current density, electrode distance and electrolysis time respectively.

### Adequacy of developed models

**Figure 2:**
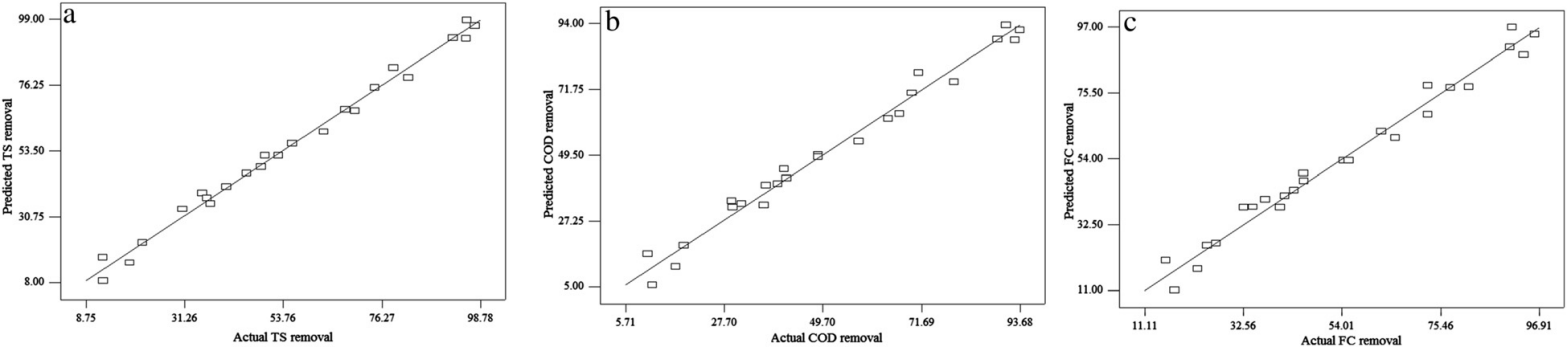
**Actual versus predicted plots for the model adequacy testing. (a)** TS removal **(b)** COD removal **(c)** FC removal.

The adequacy of models was evaluated by constructing diagnostic plots such as predicted versus actual plots for xthe experimental data obtained from this study and it is shown in Figure 2. From the Figure 2(a-c), it is observed that, the data points on this plot lie very close to the diagonal line, because residuals for the prediction of each response is minimum. Moreover, ANOVA results (Table 4) was also generated to check the adequacy of developed models which shows low CV, AP value and acceptable mean square, F- value and p-value for individual and interactive effects [20]. These results indicated a good adequate agreement between experimental data and the data predicted by the developed models.

**Table 4:** ANOVA analysis and statistical parameters of the responses

**Sources**	**TS removal**			**COD removal**			**FC removal**		
	**Mean square**	**F-value**	**p-value**	**Mean square**	**F-value**	**p-value**	**Mean square**	**F-value**	**p-value**
Model	1637.35	161.72	< 0.0001	1570.57	78.50	< 0.0001	1408.64	60.64	< 0.0001
X1	1417.45	140.00	< 0.0001	1317.34	65.85	< 0.0001	1151.50	49.57	< 0.0001
X2	3056.66	301.91	< 0.0001	2826.86	141.30	< 0.0001	2936.57	126.41	< 0.0001
X3	1683.65	166.30	< 0.0001	1386.11	69.28	< 0.0001	1161.71	50.01	< 0.0001
X4	8088.10	798.87	< 0.0001	7763.27	388.05	< 0.0001	6747.87	290.47	< 0.0001
X_1_X_2_	5.98	0.59	0.4550	0.00	0.00	0.9974	16.73	0.72	0.4104
X_1_X_3_	393.63	38.88	< 0.0001	407.43	20.37	0.0005	381.81	16.44	0.0012
X_1_X_4_	75.43	7.45	0.0163	59.06	2.95	0.1078	25.55	1.10	0.3120
X_2_X_3_	165.64	16.36	0.0012	256.00	12.80	0.0030	200.36	8.62	0.0108
X_2_X_4_	1.31	0.13	0.7243	0.62	0.03	0.8632	7.16	0.31	0.5877
X_3_X_4_	1.88	0.19	0.6733	12.25	0.61	0.4470	0.13	0.01	0.9415
X_1_^2^	5623.68	555.46	< 0.0001	5514.08	275.62	< 0.0001	4889.96	210.50	< 0.0001
X_2_^2^	1138.71	112.47	< 0.0001	1392.15	69.59	< 0.0001	1154.38	49.69	< 0.0001
X_3_^2^	1827.93	180.55	< 0.0001	1739.56	86.95	< 0.0001	1463.19	62.99	< 0.0001
X_4_^2^	3105.19	306.70	< 0.0001	3057.94	152.85	< 0.0001	2862.70	123.23	< 0.0001
Residual	10.12			20.01			23.23		
CV%	5.4			8.17			8.2		
AP	39.43			27.27			24.75		

### Effect of process variables

Three dimensional (3D) response surface plots are plotted using developed mathematical models (Equations 2,3,4) in order to study the individual and interactive effect among the process variables on the responses and also used to determine the optimal condition of each factor for maximum removal efficiency of TS, COD and FC.

### Effect of initial pH

**Figure 3:**
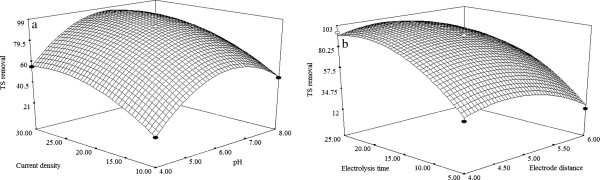
**Response surface plots (3D) for the effects of variables on the TS removal. (a)** Effect of pH and current density **(b)** Effect of electrode distance and electrolysis time.

**Figure 4:**
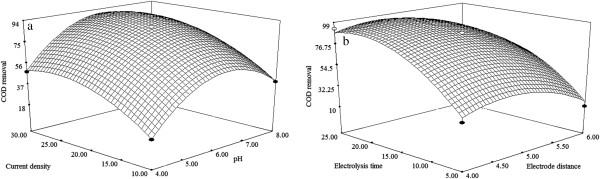
**Response surface plots (3D) for the effects of variables on the COD removal. (a)** Effect of pH and current density **(b)** Effect of electrode distance and electrolysis time.

**Figure 5:**
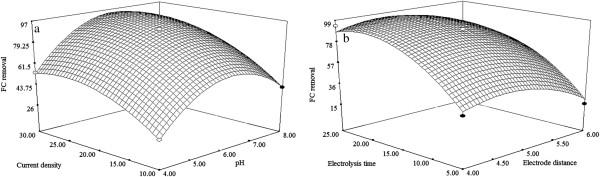
**Response surface plots (3D) for the effects of variables on the FC removal. (a)** Effect of pH and current density **(b)** Effect of electrode distance and electrolysis time.

It has been established that the intial pH is an important parameter influencing the performance of the EC process. To examine its effect, the wastewater sample was adjusted to a desired pH for each experiment by using sodium hydroxide or Hydrochloric acid. From the results (Figures 3a, 4a, 5a), it was observed that, the removal efficiency of TS, COD and FC was increased linearly with increasing initial pH from 4–6. This is mainly due to the fact that, when pH is within the range of 5.0–7.0, the formation of amorphous M(OH)_3_ species is predominant. The freshly formed amorphous M(OH)_3_ species has large surface area, which are beneficial for a removal of the TS,COD and FC via a sweep coagulation followed by precipitation mechanism. However, initial pH beyond 6 resulted in lower removal efficiencies was noticed due to the formation of monomeric M(OH)_4_^-^ species [9].

### Effect of current density

Current density is one of the important factor influence the electrocoagulation process. From the results (Figures 3a, 4a, 5a), it is found that, the removal efficiency of TS, COD and FC are increased rapidly upto current density of 20 mA/cm^2^. This is explained the fact that, the coagulant production on the anode and cathode increases while increase the current density. Therefore, there is an increase in metal hydroxide (M(OH)_3_) flocks formation in the reactor and hence theimprovement in the removal efficiencies. But, at higher current density (25–30 mA/cm^2^), the removal of TS, COD and FC are almost constant [21]. Similar results were also noticed for treatment of paper mill bleaching wastewater using EC process [9].

### Effect of electrode distance

To examine the effect of electrode distance on the EC process, experiments were conducted. From the results (Figures 3b, 4b, 5b), it is observed that, removal efficiency of TS, COD and FC are increased with the increasing electrode distance upto 5 cm. At minimum inter electrode distance the resistance for current flow in the reactor is lower that facilitates the EC process for enhanced removal of TS, COD and FC. But, beyond 5 cm of electrode distance shows the decrease in removal efficiency of TS, COD and FC. This is due to the formation of ohmic loses which in turn inhibits the production of M(OH)_3_ flocs, thus the removal of TS, COD and FC gets decreased [22].

### Effect of electrolysis time

Electrolysis time is of vital importance in the performance EC process. From the Figures 3b, 4b, 5b, it is found that, removal of efficiency of TS, COD and FC increases with increasing electrolysis time upto 15 min, thereafter removal efficiency shows almost constant. It can be explained by the fact that electrolysis time increases, an increase occurs in the amount of metal hydroxide flocs (M(OH)_3_) which promotes the removal of TS, COD and FC via a sweep coagulation followed by precipitation mechanism, thus removal effciencey of TS, COD and FC increased. Thereafter (15–30 min), almost all toxic matters are removed as flocs and hence no change in removal of TS, COD and FC removal with the increase in electrolysis time [22].

### Optimization

In order to determine the optimum process parameters for maximum TS, COD and FC removal efficiencies, Derringer’s desired function methodology optimization was used in this present study. This numerical optimization technique evaluates a point that maximizes the desirability function. According to BBD results, optimal operating conditions for the maximum removal of TS, COD and FC based on Derringer’s desired function methodology is found to be initial pH of 7, current density of 20 mA/cm^2^, electrode distance of 5 cm and electrolysis time of 20 min. Under these conditions, predicted removal efficiency of TS, COD and FC is found to be 99.87, 95.47 and 97.15% respectively. Experiments were performed under the optimized conditions, which shows the removal efficiency of TS, COD and FC close to predicted values (98.45, 94.75 and 96.34% respectively).

## Conclusions

In this study, BBD was employed to study and optimize the process variables under different operating conditions such as initial pH, current density, electrode distance and electrolysis time to treat grey wastewater by EC process using stainless steel electrode in batch mode. From the results, it was observed that, the operating variables have significant effects on the EC process. Quadratic mathamatical models were developed for predicting the removal of TS, COD and FC and optimum operating conditions was determined by Derringer’s desired function methodology. The optimal conditions were found to be: initial pH of 7, current density of 20 mA/cm^2^, electrode distance of 5 cm and electrolysis time of 20 min. Under these optimal operating conditions, the experimental removal efficiencies (98.45, 94.75 and 96.34%) was closely agreed with the predicted values (99.87, 95.47 and 97.15%). These results indicated that EC process can be scale-up in large scale level to treat grey wastewater with high removal efficiency of TS, COD and FC.

## Competing interests

The authors declare that they have no competing interests.

## Authors’ contributions

All authors read and approved the final manuscript.

## References

[B1] Yi Jing C Mei Fong C Chung Lim L Hassell DG A review on anaerobic–aerobic treatment of industrial and municipal wastewater Chem Eng J 2009 155 1 18 10.1016/j.cej.2009.06.041

[B2] Eriksson E Auffarth K Eilersen AM Henze M Ledin A Household chemicals and personal care products as sources for xenobiotic organic compounds in grey wastewater Water SA 2003 29 135 146

[B3] Elmitwalli TA Shalabi M Wendland C Otterpohl R Grey water treatment in UASB reactor at ambient temperature Water Sci Technol 2007 55 173 180 17506435 10.2166/wst.2007.142

[B4] Shin HS Lee SM Seo IS Kim GO Lim KH Song JS Pilot-scale SBR and MF operation for the removal of organic and nitrogen compounds from greywater Water Sci Technol 1998 38 79 88

[B5] Lesjean B Gnirss R Greywater treatment with a membrane bioreactor operated at low SRT and low HRT Desalination 2006 199 432 434 10.1016/j.desal.2006.03.204

[B6] Li Z Gulyas H Jahn M Gajurel DR Otterpohl R Greywater treatment by constructed wetland in combination with TiO_2_-based photocatalytic oxidation for suburban and rural areas without sewer system Water Sci Technol 2003 48 101 106 14753524

[B7] Bektas N Akbulut H Inan H Dimoglo A Removal of phosphate from aqueous solutions by electro-coagulation J Hazard Mater 2004 106 101 105 15177098 10.1016/j.jhazmat.2003.10.002

[B8] Adhoum N Monser L Bellakhal N Belgaied JE Treatment of electroplating wastewater containing Cu^2+^, Zn^2+^ and Cr (VI) by electrocoagulation J Hazard Mater 2004 B112 207 213 15302441 10.1016/j.jhazmat.2004.04.018

[B9] Sridhar R Sivakumar V Prince Immanuel V Prakash Maran J Treatment of pulp and paper industry bleaching effluent by electrocoagulation process J Hazard Mater 2011 186 1495 1502 10.1016/j.jhazmat.2010.12.028 21227578

[B10] Lai CL Lin SH Treatment of chemical mechanical polishing wastewater by electrocoagulation: system performances and sludge settling characteristics Chemosphere 2004 54 235 242 10.1016/j.chemosphere.2003.08.014 14575735

[B11] Kobya M Can OT Bayramoğlu M Treatment of textile wastewaters by electrocoagulation using iron and aluminum electrodes J Hazard Mater 2003 B100 163 178 12835020 10.1016/s0304-3894(03)00102-x

[B12] Inan H Dimoglo A Şimşek H Karpuzcu M Olive oil mill wastewater treatment by means of electro-coagulation Sep Purif Technol 2004 36 23 31 10.1016/S1383-5866(03)00148-5

[B13] Prakash Maran J Sivakumar V Thirgananasambandham K Sridhar R Microwave assisted extraction of pectin from waste Citrullus lanatus fruit rinds Carbohyd Polym 2014 101 786 791 10.1016/j.carbpol.2013.09.062 24299839

[B14] Prakash Maran J Sivakumar V Thirgananasambandham K Kandasamy S Modeling and analysis of film composition on mechanical properties of maize starch based edible films Int J Biol Macromol 2013 62 565 573 24080451 10.1016/j.ijbiomac.2013.09.027

[B15] Thirugnanasambandham K Sivakumar V Prakash Maran J Kandasamy S Chitosan based grey wastewater treatment- a statistical design approach Carbohyd Polym 2013 99 2 593 600 10.1016/j.carbpol.2013.08.058 24274548

[B16] Thirugnanasambandham K Sivakumar V Prakash Maran J Application of chitosan as an adsorbent to treat rice mill wastewater-mechanism, modeling and optimization Carbohyd Polym 2013 97 2 451 457 10.1016/j.carbpol.2013.05.012 23911470

[B17] Thirugnanasambandham K Sivakumar V Prakash Maran J Treatment of egg processing industry effluent using chitosan as an adsorbent J Serb Chem Soc doi:10.2298/JSC130201053T

[B18] Thirugnanasambandham K Sivakumar V Prakash Maran J Optimization of electrocoagulation process to treat biologically pretreated bagasse effluent-response surface analysis J Serb Chem Soc doi:10.2298/JSC130408074T

[B19] Prakash Maran J Sivakumar V Thirgananasambandham K Sridhar R Response surface modeling and analysis of barrier and optical properties of maize starch edible films Int J Biol Macromol 2013 60 412 421 23817091 10.1016/j.ijbiomac.2013.06.029

[B20] Prakash Maran J Sivakumar V Thirgananasambandham K Sridhar R Degradation behavior of biocomposites based on cassava starch buried under indoor soil conditions Carbohyd Polym 2014 101 20 28 10.1016/j.carbpol.2013.08.080 24299744

[B21] Mahesh S Prasad B Mall ID Mishra IM Electrochemical degradation of pulp and paper mill waste water COD and color removal Ind Eng Chem Res 2006 45 2830 2839 10.1021/ie0514096

[B22] Thirugnanasambandham K Sivakumar V Prakash Maran J Treatment of rice mill wastewater using continuous electrocoagulation technique: optimization and modelling J Korean Chem Soc 2013 doi:10.5012/jkcs.2013.57.6.1

